# Development of Framework for Assessing Influenza Virus Pandemic Risk

**DOI:** 10.3201/eid2108.141086

**Published:** 2015-08

**Authors:** Susan C. Trock, Stephen A. Burke, Nancy J. Cox

**Affiliations:** Centers for Disease Control and Prevention, Atlanta, GA, USA (S.C. Trock, S.A. Burke, N.J. Cox);; Battelle, Atlanta (S.A. Burke)

**Keywords:** influenza, influenza virus, viruses, risk assessment, prepandemic preparation, H7N9, H3N2v, zoonoses

## Abstract

Although predicting which influenza virus subtype will cause the next pandemic is not yet possible, public health authorities must continually assess the pandemic risk associated with animal influenza viruses, particularly those that have caused infections in humans, and determine what resources should be dedicated to mitigating that risk. To accomplish this goal, a risk assessment framework was created in collaboration with an international group of influenza experts. Compared with the previously used approach, this framework, named the Influenza Risk Assessment Tool, provides a systematic and transparent approach for assessing and comparing threats posed primarily by avian and swine influenza viruses. This tool will be useful to the international influenza community and will remain flexible and responsive to changing information.

## Abstract 2

This simple, additive, multiattribute assessment tool can evaluate the risk posed by novel influenza A viruses.

Pandemic influenza remains a formidable threat to human health. Advances in national pandemic preparedness have been made during recent decades; however, the frequent infection of humans with novel influenza viruses complicates implementation of effective control measures such as vaccines (*1*). The emergence of the influenza A(H1N1)pdm09 virus (*2*), the ongoing outbreaks of highly pathogenic avian influenza A(H5N1) viruses (*3*), and, more recently, the identification of severe human infections caused by avian influenza A(H7N9) virus in China (*4**,**5*) indicate the need for a more objective, systematic, and transparent approach for evaluating newly emerging influenza viruses with pandemic potential. The Influenza Risk Assessment Tool (IRAT) was developed in response to this need and creates a framework for systematically combining input from influenza experts to support risk management decisions that have important cost implications.

## Development of a Framework

**Figure 1:**
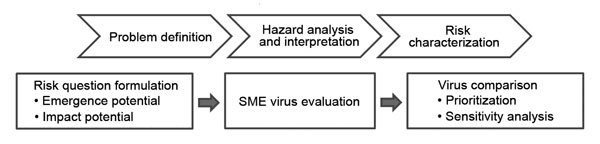
Alignment of the Influenza Risk Assessment Tool with a general microbial risk assessment framework. SME, subject matter expert.

The approach described here uses opinions from subject matter experts to populate a simple, multiattribute additive model (*6*) that combines information from well-established decision analysis methods (*7**,**8*) to assist in decision making and prioritization processes. Traditional risk assessment approaches (*9*) are not directly applicable to the IRAT; however, the general guiding principles still apply (Figure 1). The problem definition addressed by IRAT is captured in the formulation of 2 important questions. One question addresses the risk that the virus will achieve sustained human-to-human transmission and emerge as a pandemic virus (the emergence question) and specifically asks, “What is the risk that a virus not currently circulating in the human population has potential for sustained human-to-human transmission?” The second question, called the impact question, asks, “If the virus were to achieve sustained human-to-human transmission, what is the risk that a virus not currently circulating in the human population has the potential for significant impact on public health?” Risk analysis and interpretation involve the assessment of the potential pandemic risk of specific influenza viruses by subject matter experts, who use multiple factors or risk elements identified and defined by a panel of influenza experts as critical for a risk-based assessment. These defined risk elements provide the basis for evaluation and comparison of emerging influenza viruses and contribute to an aggregate risk score.

### Identification and Definition of Risk Elements

Leading influenza subject matter experts in human and animal health, along with laboratory workers, field epidemiologists, and risk modelers, were invited to a meeting in 2011 (Technical Appendix) and asked to provide input into the development of the IRAT. Participants were asked to provide and refine definitions of elements used by the IRAT to evaluate the potential risk of novel influenza viruses. To distinguish among novel influenza viruses, each element must be amenable to qualitative or quantitative evaluation, independent of other elements, and must not be redundant in what is being measured. Subject matter experts agreed on and defined 10 elements to be scored that would provide the necessary critical information. Each of the 10 elements falls into 1 of 3 categories: virus properties, attributes and immune response of the human population, and the ecology and epidemiology associated with the novel influenza virus (Table 1). These 10 elements are meant to capture the features of the virus, the population, and the ecology and epidemiology likely to affect whether or not a specific zoonotic virus can spread widely in humans. These elements are also meant to capture factors likely to affect public health during a pandemic caused by a specific virus.

**Table 1:** Risk element categories in the Influenza Risk Assessment Tool

Category	Risk element	Description
Virus properties	Genomic variation	Captures the degree of mutation and reassortment as a measure of the genetic diversity of a novel influenza virus; also captures presence or absence of known molecular markers denoting virulence
	Receptor-binding properties	Virus-binding preference to glycans with sialic acid in α-2,6 (human) linkage at the terminal galactose when compared with viruses that bind to sialic acid by α-2,3 (avian) linkage
	Transmissibility in animal models	Transmission of animal influenza viruses in >1 accepted animal models by direct contact or through respiratory droplets in the absence of direct contact
	Antiviral treatment susceptibility	Predicted or demonstrated efficacy of available (approved for human use) antiviral agents against animal influenza viruses
Host properties	Population immunity	Detection of preexisting cross-reactive serum antibodies acquired through prior infection or vaccination (examined in all age groups)
	Disease severity	Spectrum of illness with infection by a novel influenza A virus in humans or in experimentally infected animal models as surrogates for human disease
	Antigenic relationship to vaccines	Antigenic relatedness measured by hemagglutination inhibition or virus neutralization tests with postinfection ferret antiserum to emerging virus and seasonal vaccine and reference viruses
Ecology and epidemiology	Human infections	Occurrence of human infections with animal influenza viruses, frequency of these human infections, and extent of human-to human transmission of these viruses
	Infections in animals	The virus’s ability to infect animal species naturally, the number and diversity of those species, ability to maintain sustained natural transmission in those populations, and potential extent of exposure between humans and those animal species
	Global distribution (in animals)	Spatial and temporal distribution of animal influenza viruses and the effect of animal production and management systems on the spread among animal populations and potential exposure to humans

Definitions required for each element included scoring criteria that characterize viruses as low, moderate, and high risk to assist the experts in estimating a score on a scale of 1–10. For example, the definition of the element “Antivirals and Treatment Options” states, “For the purposes of the risk assessment tool, antiviral susceptibility refers to the predicted or demonstrated efficacy of available antiviral agents against animal influenza viruses.” A low-risk score (*1*–*3*) for this element is defined as “no evidence of clinically relevant resistance to any of the antiviral drugs approved for human use (neuraminidase inhibitors and M2 blockers).” A moderate-risk score (*4*–*7*) is defined as “sensitive to all neuraminidase inhibitors but resistant to M2 blockers.” A high-risk score (*8*–*10*) for this element is defined as “resistant to one or more neuraminidase inhibitor antiviral drugs.” All 10 elements have definitions for low-, moderate-, and high-risk scores.

### Weighting of Risk Elements

Because not all risk elements are equally useful in answering each risk question, a weighting system had to be determined before the final risk score for each virus was generated. A key requirement for creating a weighting system was minimizing bias regarding the weighting of input from each expert’s field of study relative to that of others and reducing the difficulty of eliciting relative weights from experts when multiple attributes are compared. A surrogate weighting method was selected for this purpose (*10*). First, the expert panel was surveyed to determine a consensus rank ordering of the 10 risk elements if each risk question was asked separately. When asked to rank the elements when the “emergence question was considered,” 27 of 29 subject matter experts agreed that evidence of human infections is the “most important” of the 10 elements regarding risk that a novel influenza virus can achieve sustained human-to-human transmission. The other 2 subject matter experts believed this element was “very important.” Second in importance was information regarding laboratory animal transmission studies; 26 subject matter experts thought this element was “most important” or “very important.” Third in importance was receptor binding information and population immunity. Of least importance for the experts was information regarding antiviral and treatment options and disease severity; 24 of the 29 subject matter experts rated this element as “not relevant” for the question about the risk that a novel influenza virus can achieve sustained human-to-human transmission. 

The second risk question addresses the potential effect of a novel influenza virus on public health in the event that the virus achieved sustained human-to-human transmission (i.e., the impact question). Subject matter experts agreed that knowledge regarding disease severity ranked first (26 ranked this element as “most important” and 3 ranked it as “very important”), followed by information pertaining to population immunity (21 ranked this element as “most important” and 8 ranked it as “very important”), human infections, and antiviral and treatment options in descending order. Of least importance were the elements related to global distribution in animal species (16 ranked this element as “not relevant”) and infections in animals (11 ranked this element as “not relevant”). Although risk element definitions were the same for the emergence and impact questions, the rank order of the risk elements by experts differed for the 2 questions.

This rank ordering of risk elements for the 2 questions facilitated the calculation of surrogate weights by using the formula

 where *K* is the number of elements and *Wk* is the calculated weight to be applied to each risk element in rank order (*10*). Thus, the weight for the top-ranked element of the 10 total elements was calculated as 0.2929, and the lowest-ranked element was assigned a weighting score of 0.001. By convention, the sum of all 10 weights in this example is 1.

### Scoring

Using the framework of risk element definitions, scoring criteria, and rank-ordered surrogate weights, individual experts were asked to evaluate viruses by using a particular risk element within their field of expertise and to provide a point estimate score. The returned scores for each risk element were averaged and then multiplied by the corresponding surrogate weight, depending on the risk question being asked. A summation of the product of all 10 risk elements provided the final aggregate score for each virus. Tables 2 and 3 show results of subject matter experts’ scoring of 3 avian influenza viruses. The H1N1 subtype virus found in North American mallards was used as an example of an influenza virus that has not infected people and appears to be wholly avian in characteristics.

**Table 2:** Risk scores and ranked weighting applied to 3 influenza viruses for the question “What is the risk that a virus not currently circulating in the human population has potential for sustained human-to-human transmission?”*

Element	Wt	HPAI H5N1 clade 1 (A/VN/1203/2004)			North American mallard influenza A(H1N1) (A/duck/NewYork/1996)			Variant H3N2 (A/Indiana/08/2011)	
		RS	Wt x RS		RS	Wt x RS		RS	Wt x RS	
Human infection	0.2929	5.67	1.66		2.33	0.68		4.33	1.27	
Transmission (laboratory animals)	0.1929	3.00	0.58		2.00	0.39		9.00	1.74	
Receptor binding	0.1429	3.30	0.47		2.00	0.29		8.30	1.19	
Population immunity	0.1096	8.67	0.95		3.00	0.33		3.67	0.40	
Infection in animals	0.0846	7.25	0.61		2.00	0.17		8.00	0.68	
Genomic variation	0.0646	4.00	0.26		3.00	0.19		8.00	0.52	
Antigenic relationship	0.0479	6.00	0.29		2.00	0.10		8.00	0.38	
Global distribution (animals)	0.0336	5.50	0.18		2.50	0.08		7.00	0.24	
Disease severity	0.0211	8.50	0.18		2.25	0.05		6.00	0.13	
Antiviral/treatment options	0.0010	4.50	0.00		2.25	0.00		2.50	0.00	
Total	1.0000		5.18			2.28			6.55	

*Wt, weight; HPAI, highly pathogenic avian influenza; RS, risk score. Weights are expressed to 4 decimal places because of the convention that the sum must be exactly 1. Sums for viruses may not be exact due to rounding.

**Table 3:** Risk scores and ranked weighting applied to 3 influenza viruses for the question “If the virus were to achieve sustained human-to-human transmission, what is the risk that a virus not currently circulating in the human population has the potential for significant impact on public health?”*

Element	Wt	HPAI H5N1 clade 1 (A/VN/1203/2004)			North American mallard H1N1 (A/duck/NewYork/1996)			Variant H3N2 (A/Indiana/08/2011)	
		RS	Wt x RS		RS	Wt x RS		RS	Wt x RS
Disease severity	0.2929	8.50	2.49		2.25	0.66		6.00	1.76
Population immunity	0.1929	8.67	1.67		3.00	0.58		3.67	0.71
Human infections	0.1429	5.67	0.81		2.33	0.33		4.33	0.62
Antiviral/treatment options	0.1096	4.50	0.49		2.25	0.25		2.50	0.27
Antigenic relatedness	0.0846	6.00	0.51		2.00	0.17		8.00	0.68
Receptor binding	0.0646	3.30	0.21		2.00	0.13		8.30	0.54
Genomic variation	0.0479	4.00	0.19		3.00	0.14		8.00	0.38
Transmission (laboratory animals)	0.0336	3.00	0.10		2.00	0.07		9.00	0.30
Global distribution (animals)	0.0211	5.50	0.12		2.50	0.05		7.00	0.15
Infections in animals	0.0010	7.25	0.01		2.00	0.00		8.00	0.01
Total	1.0000		6.60			2.38			5.42

*Wt, weight; HPAI, highly pathogenic avian influenza; RS, risk score. Weights are expressed to 4 decimal places because of the convention that the sum must be exactly 1. Sums for viruses may not be exact due to rounding.

### Addressing Uncertainty

Inherent in any risk assessment is the need to address uncertainty. The IRAT attempts to capture this factor by asking subject matter experts to characterize uncertainty in 2 ways. First, uncertainty is captured by each expert’s point estimate score on a scale of 1–10 (i.e., 1 representing the lowest level of risk and 10 representing the highest), along with an upper and lower boundary for their scores, thereby providing a range for their point scores. A second measure of uncertainty uses a confidence score from each expert; this score expresses the expert’s confidence in the available data used to make the point estimate. Finally, the subject matter experts were asked to provide a basis or justification for their risk scores. This justification could include key references and data along with professional observations and experiences. This approach was based on the assumption that subject matter experts using the same knowledge base will produce similar scores for each virus and narrow uncertainty ranges unless data are insufficient or the experts have a fundamental disagreement in the interpretation of available data. The upper and lower bounds of the score for each risk element can be used to determine the highest or lowest (respectively) risk score for a given virus. In addition, a sensitivity analysis wherein the rank order of 2 risk elements can be swapped and the risk score recalculated on the basis of the new weights can be used to show the effect of these changes on a virus’s aggregate score. The level of uncertainty is communicated in the final report of virus scores so that decision makers using the IRAT can use this information in their considerations. Even with these efforts to measure and communicate uncertainty, the numerical score produced by the IRAT may create a sense of precision that is unfounded. Anyone making decisions by using the IRAT scores needs to be reminded that the scores are semiquantitative.

## Case Illustrations

### H3N2v Virus

In July 2011, the Centers for Disease Control and Prevention (CDC; Atlanta, GA, USA) confirmed the first human case of an influenza A(H3N2) variant [A(H3N2)v] virus that had acquired the matrix gene from the A(H1N1)pdm09 virus. Eleven additional cases were reported during the rest of 2011, for a total of 12 confirmed cases in 5 states (*11*). A preliminary risk assessment using the IRAT was conducted on the basis of information gathered from these 12 cases and available laboratory data (Tables 2, 3). The H3N2v risk score for both the emergence and impact questions in December 2011 placed this virus in the moderate-risk category; the summary scores for both questions were >5.0.

**Figure 2:**
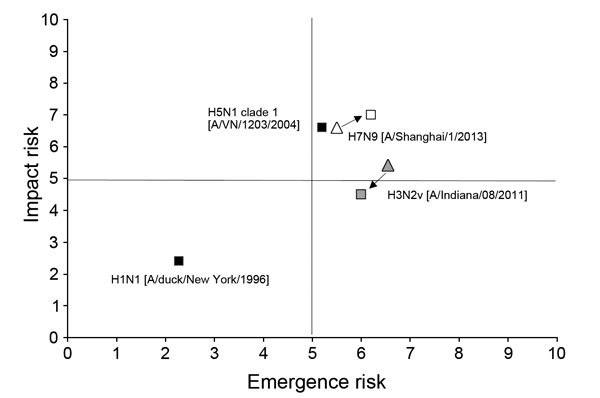
Influenza Risk Assessment Tool scores for 4 influenza viruses on the basis of potential risk to achieve “sustained human-to-human transmission” (emergence) and potential risk “for significant impact on public health” (impact). Black squares in top right quadrant and lower left quadrant represent risk scores for H5N1 clade 1 and H1N1, respectively. White triangle represents risk score for H7N9 in mid-April 2013; white square represents risk score for the same virus in mid-May 2013. Gray triangle represents risk score for H3N2v as of December 2011; gray square represents risk score for same virus in December 2012. Emergence risk is the risk summary score for the question, “What is the risk that a virus not currently circulating in the human population has the potential for sustained human-to-human transmission?” Impact risk is the risk summary score for the question, “If the virus were to achieve sustained human-to-human transmission, what is the risk that a virus not currently circulating in the human population has the potential for significant impact on public health?”

In 2012, a total of 309 cases of A(H3N2)v virus were reported from 12 states. In most cases, the putative source was direct or indirect contact with swine (*12*), with no evidence of community transmission. Studies conducted to assess population immunity (*13**–**15*) provided additional information regarding age groups considered at risk for infection. In 2013, subject matter experts rescored the H3N2v virus (Figure 2) in light of these and other available data. Although 321 confirmed cases were reported over 2 years, the summary scores for the virus were lower than those for the previous scoring. The risk score for the emergence question dropped from 6.5 to 6.1, and the risk score for the impact question dropped from 5.4 to 4.5. This reduction in the scores was caused primarily by information about population immunity. This information indicated that persons at greatest risk for infection were children, especially those <10 years of age; those 20–50 years of age exhibited cross-reactive antibodies against this virus. In addition, the epidemiologic investigations of the 309 cases in 2012 showed that >90% of the patients were <18 years of age; of these cases, 16 hospitalizations and 1 death associated with A(H3N2)v virus were documented (*16**,**17*). These epidemiologic observations reinforced the population immunity data obtained in the laboratory and supported the virus’s decreased cumulative risk score from its risk score in 2011.

### H7N9 Virus

On March 31, 2013, the China Health and Family Planning Commission notified the World Health Organization of 3 cases of human infection with influenza A(H7N9) virus (*4*). As of April 8, 2013, the China World Health Organization Collaborating Centre for Reference and Research on Influenza, the China OIE (World Organisation for Animal Health) Reference Laboratory for avian influenza, and a provincial public health laboratory in China had identified and analyzed 7 virus isolates. The complete viral genome sequences of 6 isolates were deposited in a publicly accessible influenza database, and additional human cases continued to be reported. 

In April 2013, subject matter experts used the IRAT and its 10 individual risk elements to assess the potential pandemic risk for humans posed by the novel A(H7N9) virus. To address the risk of the virus to achieve human-to-human transmission (emergence question), information about the virus’s ability to transmit in laboratory animals was critical. This information was not available in mid-April 2013. The absence of information for this and other elements affected the scoring of the risk elements. The scoring for the virus’s potential for sustained human-to-human transmission was in the moderate-risk category, approaching 5.0 on a scale of 1–10 (Figure 2). The moderate-risk score perhaps emphasized missing information in >1 high-ranking element (Transmission in Laboratory Animals). For the impact question, the risk score was in the higher end of the moderate-risk range (*4*–*7*), almost 6.0. Cautionary notes regarding interpretation of the scoring in the face of data gaps were included in the subsequent report.

A month later, in mid-May, a second scoring was conducted among CDC subject matter experts (Table 4). At this time, preliminary results of studies assessing the virus’s ability to transmit between animals in a laboratory setting were available. This information and updated information pertaining to the other elements were incorporated into the evaluation process. The risk score for the virus to achieve sustained human-to-human transmission was 6.2. This score was higher than the previous score based on the preliminary evaluation in April, although both scores were still in the moderate-risk range (*4*–*7*). The risk score for the virus’s potential to impact public health if it were to achieve sustained human-to-human transmission is 7.0, which falls in the upper limit of the moderate-risk range and which is higher than the preliminary score of ≈6.0 in mid-April (Figure 2).

**Table 4:** Average risk point scores and ranked weighting applied to risk scoring of influenza A(H7N9) virus isolate A/Shanghai/1/2013 for emergence and impact questions, April 2013 and May 2013*

Emergence question					Impact question			
Element	Weight	Average point scores			Element	Weight	Average point scores	
		Apr 2013	May 2013				Apr 2013	May 2013
Human infections	0.2929	5.0	5.0		Disease severity	0.2929	9.0	8.5
Transmission (laboratory animals)	0.1929	1.0	7.0		Population immunity	0.1929	9.0	9.0
Receptor binding	0.1429	6.7	6.3		Human infections	0.1429	5.0	5.0
Population immunity	0.1096	9.0	9.0		Antivirals/treatment options	0.1096	5.4	5.8
Infections in animals	0.0846	6.0	4.7		Antigenic relationship	0.0846	6.0	3.7
Genomic variation	0.0646	8.6	8.6		Receptor binding	0.0646	6.7	6.3
Antigenic relationship	0.0479	6.0	3.7		Genomic variation	0.0479	8.6	8.6
Global distribution (animals)	0.0336	1.0	4.7		Transmission (laboratory animals)	0.0336	1.0	7.0
Disease severity	0.0211	9.0	8.5		Global distribution (animals)	0.0211	1.0	4.7
Antivirals/treatment options	0.001	5.4	5.8		Infections in animals	0.001	6.0	4.7
Weighted IRAT aggregate score		5.2	6.2		Weighted IRAT aggregate score		7.1	7.0

*IRAT, Influenza Risk Assessment Tool.

During both rounds of scoring, the subject matter experts were asked to grade their level of confidence in the available data as it applied to their scoring. They generally expressed a greater confidence in their risk scores in May. For most elements, the risk scores differed little from those in the first round. However, in May, preliminary data were available for the risk element Transmission in Laboratory Animals and informed the risk score for this element. Although the 2 scorings took place only 1 month apart, this change illustrates how a risk score can increase after availability of new data and emphasizes the necessity of reevaluating risk scores when new data become available.

## Discussion

The IRAT is not a tool to assess widespread human seasonal influenza viruses that are already able to transmit from person to person; those viruses are more correctly assessed in terms of disease severity on a season-by-season basis (*18*). For seasonal influenza viruses, decisions have already been made regarding mass manufacturing and distribution of seasonal influenza vaccines. The IRAT assesses influenza viruses that are not yet readily transmissible person to person for their potential risk of emergence and potential effect on the public’s health. Furthermore, the IRAT was not developed to predict the next pandemic influenza virus but rather to focus limited pandemic preparedness resources on those viruses that are believed, on the basis of current knowledge, to have the greatest potential to cause a serious pandemic.

Developing an accepted, systematic method for evaluating potentially pandemic influenza viruses to inform risk management decisions requires a framework capable of combining multiple data inputs and information. Conceptually, the IRAT follows the basic principles of microbial risk assessment frameworks but diverges from traditional methods. Microbial risk assessments typically identify and establish a working model that facilitates the analysis of dose responses and exposure estimates so that risks can be calculated and expressed in terms of likelihood, type, or magnitude. The IRAT focuses on a virologic assessment and prioritization. The development of the IRAT followed a process by which the components of a multiattribute decision analysis method were adapted to the application of evaluating influenza viruses with pandemic potential. For example, the IRAT can provide subject matter expert consensus input into decisions regarding whether to stockpile a particular influenza vaccine antigen in case it is needed. Although the IRAT can offer input into such a decision, it is not the only input.

Subject matter experts were called on to characterize, define, and capture the elements to consider when the IRAT was created. These subject matter experts, who generously brought years of experience, expertise, and familiarity with the published literature to the development of the IRAT, continue to play an integral part in its use and development. Beyond what is published, such experts in the global influenza community have insights regarding ongoing studies and research that remain unpublished. The use of subject matter experts to provide input into the IRAT is critical for addressing the inherent data gaps and uncertainty in the available information regarding emerging influenza viruses. The 2 case illustrations show how new information is readily incorporated into the analysis and reflected in the risk assessments.

This tool can also be used to guide global investments in capacity building and to highlight information gaps that can help focus research initiatives to fill these gaps. Such use was seen in the consideration of the H3N2v subtype virus, wherein studies exploring population immunity provided insight into age groups at risk and lowered the risk score of the virus once that information gap was filled. Although prediction of the next pandemic influenza virus is not yet possible, a systematic evaluation of emerging influenza A viruses with the IRAT has provided a more orderly prioritization of work on novel influenza viruses and a more judicious use of resources.

Missing information is clearly a vulnerability of the IRAT, as with any other risk assessment tool and as illustrated in the initial assessment of H7N9. Although some information may remain missing, the tool as designed incorporates weighting factors assigned to each element. To maximize the usefulness of the IRAT, information relating to the top-ranked elements, which carry the most weight, is the most helpful because these elements provide a greater proportion of the total risk score for a virus. However, even with missing or limited information, the IRAT can still provide useful insights about the potential risk posed by a novel influenza virus, particularly if the missing information pertains to lower-weighted elements.

As it is envisioned, the IRAT will continue to evolve. Current definitions of the elements are considered to be working definitions to enable users to familiarize themselves with the tool and its applications. As technology advances, the elements likely will change to reflect increasing understanding of influenza viruses. We hope that this tool will prove useful to the international influenza community yet remain flexible and responsive to increases in knowledge, understanding, and interpretation of potential risks posed by novel influenza viruses to human health.

**Technical Appendix.** Executive summary of meeting for Influenza Risk Assessment Algorithm Tool, October 18–19, 2011, Alexandria, VA, USA.
