# Supplementary material for: Development of Framework for Assessing Influenza Virus Pandemic Risk
Source: Emerg Infect Dis. 2015 Aug;21(8):1372–8. doi: 10.3201/eid2108.141086 (PMC4517742; doi:10.3201/eid2108.141086)
Supplement: Supplementary file 1 — Technical Appendix. Executive summary of meeting for Influenza Risk Assessment Algorithm Tool, October 18–19, 2011, Alexandria, VA, USA. [file 14-1086-Techapp-s1.pdf]

Article DOI: http://dx.doi.org/10.3201/eid2108.141086

Development of Framework for Assessing Influenza Virus Pandemic Risk

Technical Appendix

Executive Summary of Meeting for Influenza Risk Assessment Algorithm Tool, October 18–19, 2011, Alexandria, VA, USA

Introduction

The emergence of the A(H1N1)pdm09 virus and the ongoing panzootic of H5N1 subtype virus have underscored the need for a systematic and transparent approach to evaluating influenza viruses with pandemic potential to better inform decisions related to preparedness initiatives. Such an approach could also guide research needs and resource allocation. Although predicting the next pandemic virus is not yet possible, a systematic evaluation of novel, circulating influenza A viruses would enable the logical prioritization of countermeasures and judicious use of resources. The concept of an Influenza Risk Assessment Tool (IRAT) is a response to this need and would capitalize on previous global investments in capacity building. Such a tool would also highlight information gaps and could therefore drive research initiatives to fill these gaps.

The development of an IRAT was previously proposed at the “One Flu” Strategic Retreat held in Treviso, Italy, in 2011 as one of several activities intended to move the “One Flu” concept forward. Development of such a tool would motivate sharing of information and data across disciplines (e.g., animal and human health agencies, epidemiology, and laboratory).

A meeting of international participants representing various disciplines, organizations, and institutions involved in combating influenza was convened October 18–19, 2011, in Alexandria, Virginia, USA, to invite comment and input regarding the development of an IRAT. The meeting brought together leading international influenza researchers and scientists who contributed their expertise and experience. The meeting goals were to 1) introduce a framework for the IRAT and a working draft of risk elements identified by a Centers for Disease Control

and Prevention (Atlanta, GA, USA) working group, 2) solicit feedback and input to the framework and derive definitions and relative rankings of relevant attributes of influenza viruses, and 3) recruit persons from various disciplines for future participation in the development of the tool.

Meeting Proceedings

Rationale and Framework for the Creation of an Influenza Risk Assessment Tool

A Call for a Systematic Approach

Meeting participants were presented with a rationale for the development of an IRAT that primarily focused on facilitating comparisons and prioritization of prepandemic influenza A viruses in a systematic and transparent way. Although the method should be as simple and understandable as possible, the tool must be able to appropriately integrate multiple criteria or factors assigned different levels of importance and address missing or incomplete data. The final tool should be able to combine the assessment of several different measurements or elements into a single summary output to enable comparison of viruses. The tool must be flexible and enable new information to be easily incorporated into the algorithm and output. The tool is a semiquantitative method that relies on participation from experts to provide the framework and data input.

Description of the Draft Influenza Risk Assessment Tool

To create an algorithm to assess the risk of influenza viruses systematically, the meeting participants focused on the attributes, characteristics, or properties of either the virus or the host that should be considered when evaluating the risks posed by viruses that may have pandemic potential. A draft assessment framework, its methods, and several risk elements were presented and explained at the meeting. The methods followed an adaptation of a common multiattribute decision analysis tool. The meeting participants were asked to assist in developing the tool for which expert input is critical to identifying and defining relevant risk elements that provide the basis for comparing and discriminating between influenza viruses. Risk elements are rank ordered by importance according to a given risk situation so that weights can be applied to the assessment of individual viruses by panels of subject matter experts. The resultant summary score would provide the basis to compare the level of risk posed by each virus. The exact

summary scores for each virus are less important than the relative scoring that enables a comparison of viruses to each other. Such a transparent mechanism would also point out knowledge gaps needing to be addressed by additional research efforts. Viruses that score comparatively low in terms of risk may not be the subject of additional studies, whereas viruses scoring in the highest risk group would demand further investment of resources to fill information gaps. Such relative comparisons could also serve as the basis for choosing which viruses should be selected for creating high-growth vaccine candidates, manufacturing vaccines, and proceeding to clinical trials – all as components of prepandemic preparedness for viruses posing the greatest threat to public health.

Identification of Risk Elements

For the tool to provide maximum usefulness, all elements that should be incorporated into the tool are present, and the total number of elements is minimized. In brief, the criteria for the risk elements are as follows:

a. The elements, *in toto*, must capture the core considerations used in the evaluation of a prepandemic influenza A viruses, and

b. Each element can be evaluated either qualitatively or quantitatively, and

c. Each element can be assessed independently of other elements in the Tool, and

d. Each element is an important consideration when assessing a virus, and

e. An element is not duplicative of another element or elements.

The draft framework considered 10 risk elements associated with influenza viruses. Broadly, these 10 elements can be categorized into 3 major areas; 1) properties of the virus, 2) attributes of the population, and 3) the ecology and epidemiology of the virus. The 10 draft risk elements were presented, and the meeting participants further refined and defined them. Consensus was reached that these 10 elements captured the essential information necessary to differentiate prepandemic viruses from each other.

Ranking the Risk Elements

All risk elements are not equally important when considering a given situation or risk question. Therefore, each element is assigned a weight. Application of weights to the elements is preceded by determining a rank order, such that the highest-ranked risk element would be given

a greater weight in the analysis than the other elements. Similarly, the remaining ranked elements would be assigned successively lesser weights than the top-ranked element.

Once a consensus was reached regarding the definitions and descriptions of the risk elements of the tool, it was possible to rank each element in relative importance when compared to the other 9 elements. Two situations or questions were posed to the meeting participants. For each situation, the group was tasked with ranking each of the 10 elements from most important to least important. The first situation addressed the question regarding virus emergence: What is the risk that a virus not currently circulating in the human population has potential for sustained human-to-human transmission? The second situation, dealing with impact, posed the following question: If the virus were to achieve sustained human-to-human transmission, what is the risk that a virus not currently circulating in the human population has the potential for significant impact on public health? Regardless of the situation or question posed, the definition of each element did not change.

For each question, the participants were asked to consider each element, pick the single most important element that would help them answer the question, and rank it the highest. Participants then repeated the process for the remaining nine elements and continued removing 1 element at a time until they had ranked each element on a scale of 1–10. As would be expected, there was not 100% agreement on how each element should be ranked; however, there was general consensus as to which elements would be ranked highest (i.e., rank 1, 2, or 3), which would rank lowest (i.e., rank 8, 9, or 10), and which would default into the middle group. Broadly, these could be considered high-, low-, and moderate-risk categories, respectively. In addition, depending upon the situation or question asked, the elements changed rank order.

Further Development of the IRAT

Several additional steps were required to bring the development of the tool to completion. Subsequent discussion and a survey of participants indicated a high level of interest in continuing the process. Besides the technical development of the tool, it was agreed that there was a need to broaden the participation of experts and familiarize additional groups and organizations with the potential uses and limitations of the IRAT. It was agreed that it would be useful to convene a larger meeting to evaluate the IRAT after the additional refinements were made.

Observations and Issues Identified by Participants

1. Definitions and terminology are important to the interpretation and use of any assessment tool. Meeting participants noted the need for consistency in the basic format for each risk element definition and its criteria, and significant time was spent refining the definitions and striving for clarity across the multiple technical disciplines.

2. Assumptions and limitations of the specific multiattribute decision analysis methodology were discussed and noted. The assumption of independence of each element was discussed and carefully considered when definitions were refined and criteria were assigned to particular elements.

3. The uncertainty associated with lack of data or information regarding particular viruses requires a set of decision rules. Further development of the IRAT will address aspects of the methodology.

Influenza Risk Assessment Algorithm Meeting, October 18–19, 2011, Alexandria, Virginia, USA

Participant List

Mona Ali
Animal Health Research Institute, Egypt

Jill Banks
Animal Health Veterinary Laboratories Agency, United Kingdom

Ian Barr
World Health Organization Collaborating Center for Reference and Research on Influenza, Australia

Martin Beer
Friedrich-Loeffler-Institute, Germany

Rick Bright
Office of the Assistant Secretary for Preparedness and Response/US Department of Health and Human Services, USA

Stephen Burke
Battelle, Centers for Disease Control and Prevention, USA

Ilaria Capua
Istituto Zooprofilattico Sperimentale delle Venezie, Italy

Hualan Chen

Harbin Veterinary Research Institute, China

Richard Compans
Emory University, USA

José Cortiñas Abrahantes
European Food Safety Authority, Italy

Nancy Cox
Centers for Disease Control and Prevention, USA

Gwen Dauphin
Food and Agriculture Organization

Marco De Nardi
Istituto Zooprofilattico Sperimentale delle Venezie, Italy

Armen Donabedian
Office of the Assistant Secretary for Preparedness and Response/US Department of Health and Human Services, USA

Ruben Donis
Centers for Disease Control and Prevention, USA

Lyn Finelli
Centers for Disease Control and Prevention, USA

MaryKathleen Glynn
World Health Organisation for Animal Health, France

Diane Gross
Centers for Disease Control and Prevention, USA

Per Have
European Food Safety Authority, Italy

Andrew Hill
Animal Health Veterinary Laboratories Agency, United Kingdom

Jackie Katz
Centers for Disease Control and Prevention, USA

John McCauley
National Institute for Medical Research, United Kingdom

Arnold Monto

University of Michigan, USA

Liz Mumford
World Health Organization, Switzerland

Tung Nguyen
MARD National Center for Veterinary Diagnostics, Vietnam

Angus Nicoll
European Center for Disease Prevention and Control, Sweden

Dan Normandeau
Conversart, Canada

Jim Paulson
The Scripps Institute, USA

Malik Peiris
University of Hong Kong, Hong Kong

Daniel Perez
University of Maryland, USA

Julio Pinto
Food and Agriculture Organization, Italy

Diane Post
National Institute of Allergy and Infectious Diseases/National Institutes of Health, USA

Steve Redd
Centers for Disease Control and Prevention, USA

Michael Shaw
Centers for Disease Control and Prevention, USA

Kanta Subbarao
National Institute of Allergy and Infectious Diseases/National Institutes of Health, USA

David Swayne
Agricultural Research Service/US Department of Agriculture, USA

Sabrina Swenson
Animal and Plant Health Inspection Service/ US Department of Agriculture, USA

Masato Tashiro
National Institute of Infectious Diseases, Japan

Ralph Tripp
University of Georgia, USA

Susan Trock
Centers for Disease Control and Prevention, USA

Tim Uyeki
Centers for Disease Control and Prevention, USA

Amy Vincent
Agricultural Research Service/US Department of Agriculture, USA

Richard Webby
St. Jude Children's Hospital, USA

Robert Webster
St. Jude Children's Hospital, USA
